# Establishment and Evaluation of a Multicolor Latex Microsphere‐Based Lateral Flow Immunoassay for the Simultaneous Detection of Antibodies Against African and Classical Swine Fever Viruses

**DOI:** 10.1155/tbed/5512419

**Published:** 2026-01-16

**Authors:** Jie Chen, Zhengwang Shi, Yi Ru, Juncong Luo, Qianqian Yang, Yage Xie, Lin Wang, Jing Zhou, Xiaoyang Zhang, Juanjuan Wei, Yuqian Zhu, Hong Tian, Haixue Zheng

**Affiliations:** ^1^ State Key Laboratory of Animal Disease Prevention and Control, College of Veterinary Medicine, Lanzhou University, Lanzhou Veterinary Research Institute, Chinese Academy of Agricultural Sciences, Lanzhou, 730000, China, caas.cn; ^2^ Gansu Province Research Center for Basic Disciplines of Pathogen Biology, Lanzhou, 730046, China

**Keywords:** African swine fever (ASF), classical swine fever (CSF), lateral flow immunoassay (LFIA), latex microsphere (LM), point-of-care testing (POCT)

## Abstract

African swine fever (ASF), a highly fatal disease often termed the “number one killer” of pigs, presents clinical symptoms indistinguishable from classical swine fever (CSF), such as fever, diarrhea, and vomiting, complicating on‐site differential diagnosis. As both ASF and CSF are notifiable diseases under the World Organisation for Animal Health (WOAH), rapid and accurate identification is crucial for effective outbreak management. In this study, we developed a multicolor lateral flow immunoassay (LFIA) based on latex microspheres (LMs) for the simultaneous detection of antibodies against ASF virus (ASFV) and CSF virus (CSFV). The assay enables visual differentiation within 15 min, with red indicating ASFV antibodies and blue indicating CSFV antibodies. After optimization, the LFIA demonstrated a sensitivity of 1:256, equivalent to that of a commercial ASFV ELISA kit and four‐fold higher than that for CSFV (1:64). The assay exhibited high specificity, showing no cross‐reactivity with other common swine pathogens and bovine viral diarrhea virus (BVDV). When applied to 180 clinical serum samples and compared with commercial ELISA kits, the LFIA achieved Cohen’s kappa values of 0.986 for ASFV and 0.918 for CSFV, indicating excellent agreement. Additionally, intra and interbatch evaluations confirmed its robust repeatability. Overall, the multicolor LM‐LFIA offers a rapid, sensitive, specific, and cost‐effective tool for point‐of‐care testing (POCT) of ASFV and CSFV antibodies, holding promise for routine field surveillance and disease control.

**Summary**

• Visually Intuitive: African swine fever (ASFV) and classical swine fever virus (CSFV) antibodies can be simultaneously distinguished in a single assay within 15 min, indicated by distinct red (ASFV) and blue (CSFV) test lines.
• High Diagnostic Accuracy: The assay achieved Cohen’s kappa values of 0.986 and 0.918 compared to commercial ASFV and CSFV ELISA kits, respectively, indicating excellent agreement and suitability for point‐of‐care testing (POCT).
• Enhanced Sensitivity: The assay exhibited a detection limit of 1:256, matching the performance of a commercial ASFV ELISA and surpassing that of the CSFV ELISA (1:64).

## 1. Introduction

African swine fever (ASF), caused by the ASF virus (ASFV), is a highly contagious and often fatal hemorrhagic disease in pigs [1], with mortality rates reaching up to 100% [2]. Since its emergence in China in August 2018, ASF has had devastating effects on the national pig industry, leading to a 40% decline in the pig population and a significant increase in pork prices in just 1 year [3]. The global transmission of ASF is fearful, with outbreaks occurring on five continents in recent years [4, 5], which further exacerbating the global crisis. Currently, Genotype II and Genotype I/II recombinant strains predominate in China [6], and the diversity in genotypes contributes to variations in virulence and clinical presentation, complicating prevention and control strategies. ASFV is a large, icosahedral, cytoplasmic double‐stranded DNA virus classified under the genus *Asfivirus* in the Asfarviridae family. It encodes more than 150 proteins, among which the major capsid protein p72 (encoded by the *B646L* gene) is a key diagnostic target [7, 8].

Classical swine fever (CSF), another economically important transboundary disease, is caused by the CSF virus (CSFV), a single‐stranded RNA virus belonging to the *Pestivirus* genus of the Flaviviridae family [9]. Clinical manifestations of CSF are often indistinguishable from ASF [10, 11], including high fever, anorexia, lethargy, hemorrhagic lesions, and lymphadenopathy. Among its structural proteins, E2 is the most immunogenic and is the principal target for both vaccine development and serological diagnosis [12].

Currently, no effective commercial vaccines or antiviral treatments are available for ASF due to its complex structure and the limited understanding of the gene’s functions [13]. Therefore, ASF control relies heavily on rapid detection, culling of infected animals, and strict biosecurity measures [14]. In contrast, CSF can be effectively controlled through vaccination [15], particularly with the widely used live attenuated C‐strain vaccine [16]. However, vaccine efficacy may be compromised by immunosuppressive coinfections, poor vaccine quality, or improper vaccination schedules in areas where CSF is prevalent or reemerging, which means that there is still a long way to go to eradicate CSF worldwide completely [17].

Given the overlapping clinical signs and the possibility of coinfection in ASF and CSF [18], accurate differential diagnosis is essential for timely intervention and containment. Laboratory confirmation using PCR [19], LAMP [20–22], or ELISA [23–27] remains the gold standard, but these methods are time‐consuming, require specialized equipment, and are unsuitable for on‐site diagnosis, especially in resource‐limited settings. While multiplex nucleic acid‐based assays have been developed for ASFV and CSFV detection [28–31], they are not ideal for field applications due to complexity and cost. Similarly, antibody based multiplex detection methods for ASF and CSF already exist [32, 33] but often face limitations in portability and visual interpretation.

Lateral flow immunoassay (LFIA) offers a promising alternative, providing rapid, simple, and cost‐effective point‐of‐care testing (POCT). Latex microsphere (LM)‐based LFIA, in particular, offers higher sensitivity and color versatility compared to conventional colloidal gold‐based strips [34–36]. Multicolor LMs enable simultaneous detection of multiple targets with distinct visual readouts [37], making them ideal for differentiating ASFV and CSFV antibodies in a single test.

In this study, we developed a novel multicolor LM‐LFIA platform capable of simultaneously detecting ASFV and CSFV antibodies with high sensitivity, specificity, and ease of use. We evaluated its performance using standard and clinical samples and benchmarked it against commercial ELISA kits. It will offer a rapid, sensitive, specific, and cost‐effective tool for POCT of ASFV and CSFV antibodies, holding promise for routine field surveillance and disease control.

## 2. Materials and Methods

### 2.1. Materials and Instruments

LMs were obtained from Vdo Biotech Co. (Suzhou, China). 2‐(N‐morpholino) ethanesulfonic acid (MES), N‐hydroxysuccinimide (NHS), 1‐ethyl‐3‐(3‐dimethylaminopropyl) carbodiimide (EDC), bovine serum albumin (BSA), Tween‐20, and casein were purchased from Sigma‐Aldrich (Shanghai, China). Nitrocellulose (NC) membranes were sourced from Merck Millipore (Germany), while sample pads, conjugate pads, absorbent pads, PVC backing cards, and plastic housings were supplied by Jiening Biotechnology Co. (Shanghai, China). Chicken IgY (CIgY) and goat anti‐chicken IgY (GCIgY) antibodies were obtained from SolarBio (Beijing, China). BCA protein assay kit were sourced from Tiangen Biotechnology (Beijing, China). Commercial ELISA kits for ASFV antibody detection (ASF.K001/5, Ingenasa, Spain) were purchased from Qingdao RealVet Bio‐Technology Co., Ltd. (Qingdao, China), and CSFV ELISA antibody test kit (CSFV Ab) (99‐43220, IDEXX, USA) were provided by IDEXX Laboratories, Inc.

Standard ASFV‐ and CSFV‐positive and ‐negative sera, along with 180 clinical serum samples, were provided by the African Swine Fever Regional Laboratory of China (Lanzhou). Positive sera for porcine reproductive and respiratory syndrome virus (PRRSV), pseudorabies virus (PRV), porcine epidemic diarrhea virus (PEDV), porcine circovirus type 2 (PCV‐2) and bovine viral diarrhea virus (BVDV) were obtained from the Lanzhou Veterinary Research Institute (LVRI), Chinese Academy of Agricultural Sciences (CAAS). Positive sera for foot‐and‐mouth disease virus serotype O (FMDV‐O) were provided by the World Organisation for Animal Health (WOAH)/National Foot‐and‐Mouth Disease Reference Laboratory.

Key instruments included a pH meter (Mettler Toledo, Switzerland), a particle size analyzer (Malvern Instruments, UK), a BioDot XYZ3050 three‐dimensional dispenser, and a BioDot CM4000 strip cutter (Bio‐Dot Scientific Equipment, China).

### 2.2. Expression and Identification of Truncated p72 and E2 Proteins

A truncated segment of the ASFV *B646L* gene (amino acids 20–303) [38–40] and a truncated E2 gene segment from CSFV (amino acids 690–879) [41] covering major antigenic epitopes were codon‐optimized and synthesized by Sangon Biotech (Shanghai, China). These sequences were cloned into the pET‐28a and pET‐30a expression vectors, respectively, and transformed into *Escherichia coli* BL21 (DE3) competent cells. Protein expression was induced with 0.1 mM IPTG at 37°C for 8 h. Recombinant proteins were purified using Ni‐NTA affinity chromatography, analyzed by SDS‐PAGE, and quantified using a BCA protein assay kit (Tiangen Biotechnology, China). Antigenicity was validated via western blotting and indirect ELISA using standard ASFV‐ and CSFV‐positive and ‐negative sera.

### 2.3. Preparation of Immunoprobes: p72@RLM, E2@BLM, and CIgY@BkLM

Red (R), blue (B), and black (Bk) LM were conjugated with p72, E2, and CIgY proteins, respectively, to prepare visually distinguishable immunoprobes. Briefly, LMs were suspended in cold MES buffer (pH 6.2) containing 10 mg/mL NHS and 10 mg/mL EDC for activation of carboxyl group. The desired amount of each protein was then added, followed by incubation for 1 h. Unreacted groups were blocked using a blocking buffer, and the labeled LMs were centrifuged and resuspended in storage buffer (pH 7.2). All probes were stored at 4°C until use.

### 2.4. Assembly of the Multicolor LM‐LFIA

The prepared probes (p72@RLM, E2@BLM, and CIgY@BkLM) were premixed and dispensed onto the conjugate pad. On the NC membrane, 1.0 μL/cm of p72, E2, and GCIgY were immobilized sequentially to form the Test (T)1 line (ASFV), T2 line (CSFV), and control (C) line, respectively. After drying, the membrane was assembled with the conjugate pad, sample pad, and absorbent pad on a PVC backing card, with 2.5 mm overlaps. The assembled cards were then cut into 4‐mm‐wide strips and packaged in plastic cassettes for storage.

### 2.5. Optimization of Parameters

Standard positive and negative sera were diluted 10‐fold in PBS and used as controls. The color signal was observed 10 ± 3 min after sample application. For probe optimization, LMs were conjugated with different concentrations of proteins (12.5–100 μg/mL), and sedimentation was assessed at 5 min and 24 h postreaction (The method has been slightly improved based on related studies [36]). Besides, T‐line and C‐line concentrations, as well as mixing ratios of probe, were systematically optimized (data not shown).

### 2.6. Evaluation of Assay Performance

Sensitivity: Serial two‐fold dilutions (1:2 to 1:1024) of standard positive sera were tested using the developed LFIA and commercial ELISA kits. The lowest dilution yielding a visible T‐line was recorded as the detection limit. All tests were performed in triplicate. Specificity: The assay’s specificity was tested using ASFV‐ and CSFV‐positive and ‐negative sera, along with sera positive for PRRSV, PRV, PEDV, PCV‐2, BVDV, and FMDV‐O. Each condition was tested in triplicate. Repeatability: intrabatch and interbatch repeatability were assessed by testing the same serum samples using strips from the same or different production batches. Each test was repeated three times.

### 2.7. Application to Clinical Samples and Comparison With Commercial ELISA Kits

A total of 180 clinical serum samples were tested with the developed multicolor LM‐LFIA and corresponding commercial ELISA kits for ASFV and CSFV antibodies. Agreement between methods was assessed using Cohen’s kappa coefficient.

### 2.8. Statistical Analysis

Data visualization and statistical analysis were conducted using GraphPad Prism 8.0 (GraphPad Software, USA) and OriginPro 2024b (OriginLab, USA).

## 3. Results

### 3.1. Expression and Identification of Truncated p72 and E2 Proteins

The truncated p72 and E2 proteins were successfully expressed in *E. coli* and purified with high yield and purity. SDS‐PAGE analysis revealed bands corresponding to the expected molecular weights of approximately 34 kDa for p72 and 27 kDa for E2 (Figure 1A,D). Western blotting confirmed the specific reactivity of each recombinant protein with the corresponding positive sera, while no signal was detected in negative controls (Figure 1B,E). Indirect ELISA further validated the immunoreactivity and antigen specificity of both proteins, with strong positive signals and negligible background in negative samples (Figure 1C,F).

Figure 1Identification of p72 and E2 protein. (A,D) SDS‐PAGE analysis. (B,E) Western‐blot analysis. (C,F) Indirect ELISA results of proteins and standard sera. lane M, protein marker; Lane 1, p72 protein; Lane 2, p72 protein with ASFV positive serum; Lane 3, p72 protein with ASFV negative serum; Lane 4, E2 protein; Lane 5, E2 protein with CSFV positive serum; Lane 6, E2 protein with CSFV negative serum.

**Figure:**
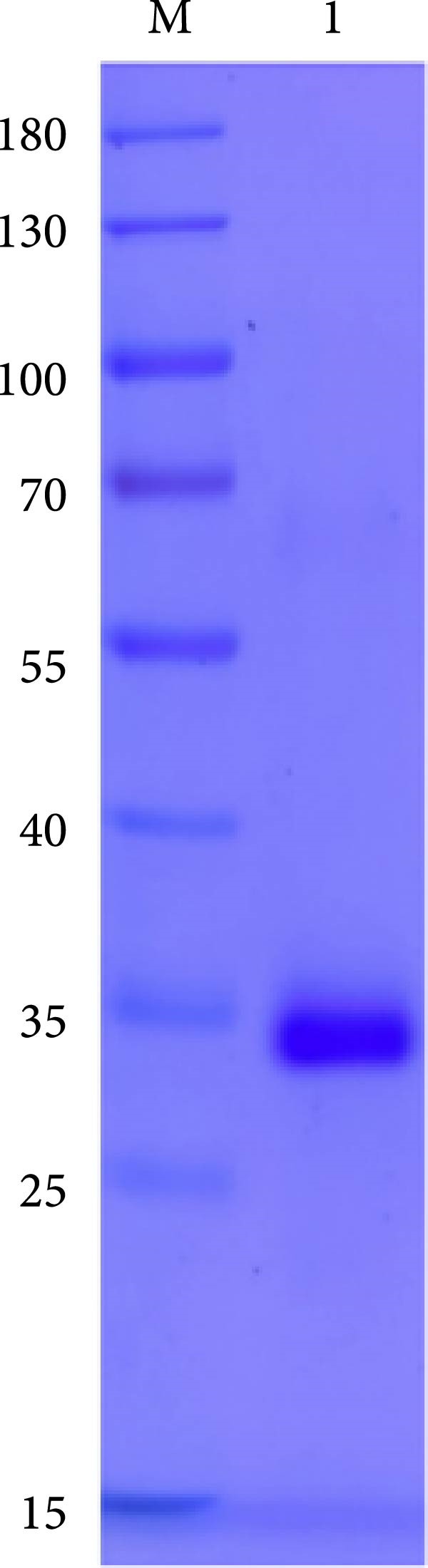
(A)

**Figure:**
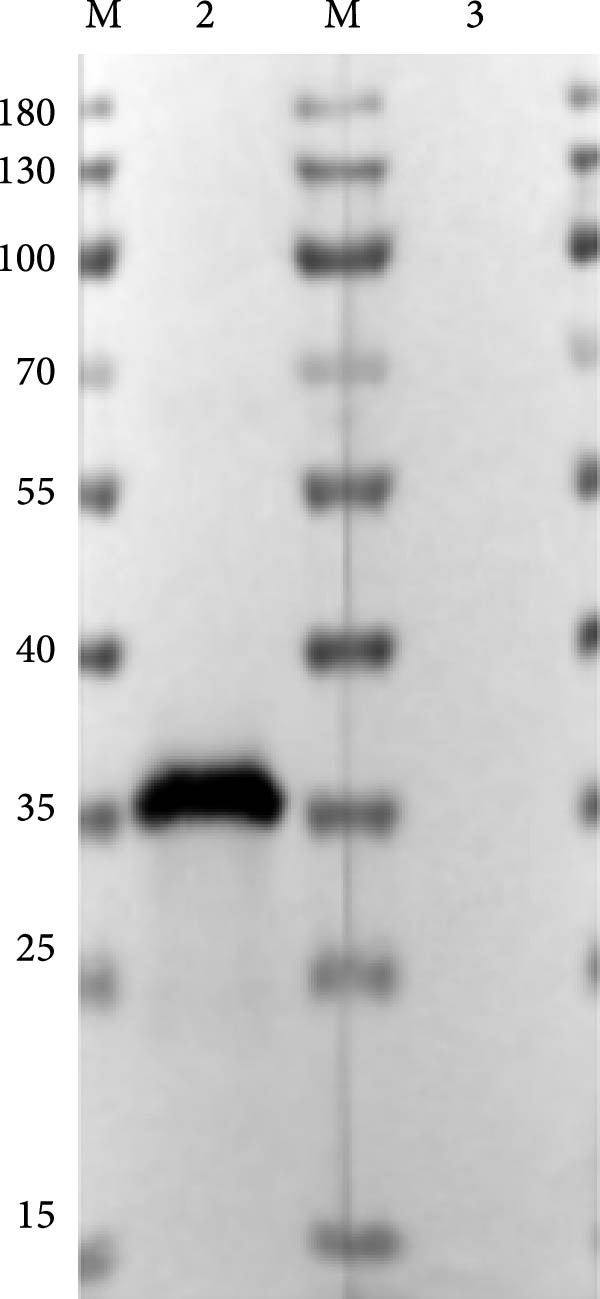
(B)

**Figure:**
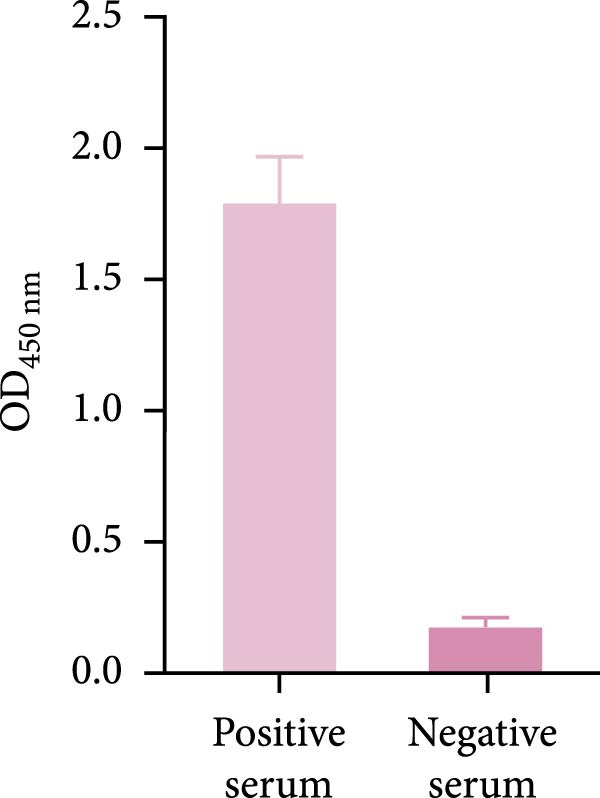
(C)

**Figure:**
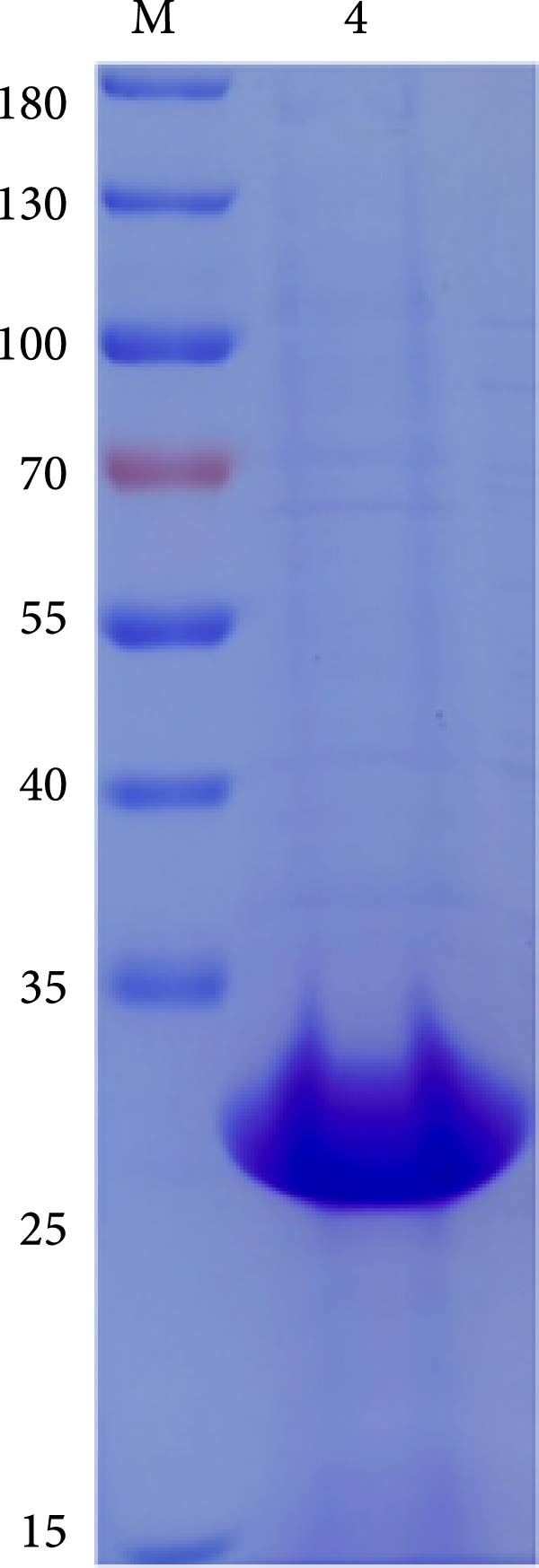
(D)

**Figure:**
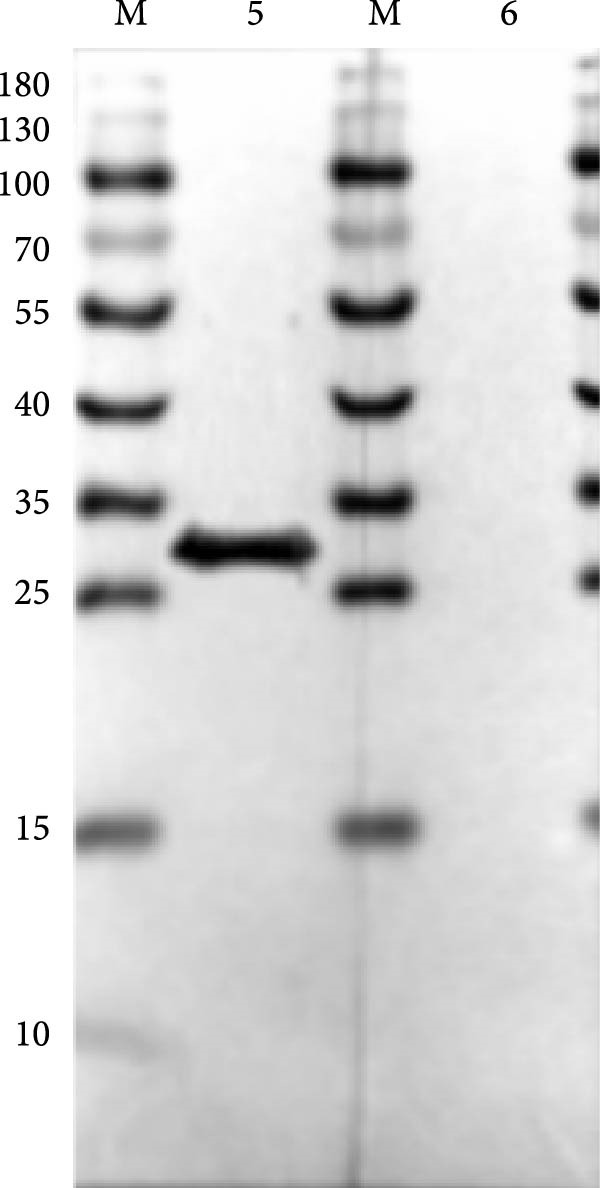
(E)

**Figure:**
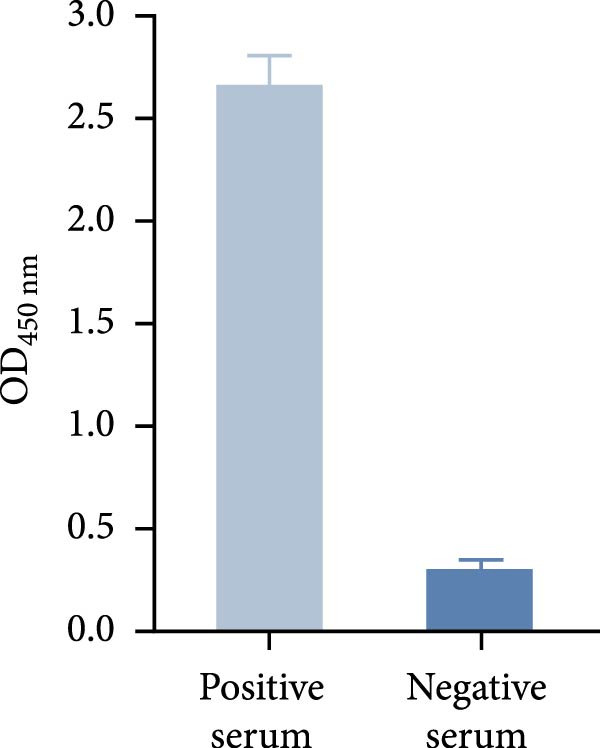
(F)

### 3.2. Mechanism of the Multicolor LM‐LFIA

The assay design is illustrated in Figure 2, in which Red (R), blue (B), and black (Bk) LMs were conjugated with p72, E2, and CIgY proteins, respectively, via EDC/NHS activation to yield immunoprobes named p72@RLM, E2@BLM, and CIgY@BkLM (Figure 2A). These probes were then deposited onto the conjugate pad, while on the NC membrane, p72, E2, and GCIgY were immobilized to form the T1‐line (ASFV), T2‐line (CSFV), and C‐line (control), respectively (Figure 2B). Upon application of the serum sample, capillary flow enables interaction between antibodies in the sample and the color‐coded probes. In the presence of ASFV‐specific antibodies, p72@RLM complexes are captured at the T1‐line, forming a visible red band. Similarly, CSFV‐specific antibodies form complexes with E2@BLM, producing a blue band at the T2‐line. CIgY@BkLM serves as an internal control and binds to the GCIgY at the C‐line to yield a black band. A valid test strip must display the C‐line, and presence of T1 and/or T2 lines indicates ASFV, CSFV, or dual antibody positivity, respectively, while absence of the T‐lines signifies a negative sample. If the C‐line is absent, the result is considered invalid (Figure 2C).

Figure 2Mechanism of the multicolor LM‐LFIA. (A) Preparation of immune probes. The carboxyl groups on the surfaces of BkLM, RLM, and BLM were activated by NHS and EDC, and then conjugated with CIgY, p72 and E2, yielding immunoprobes termed CIgY@BkLM, p72@RLM and E2@BLM, respectively. (B) Assembly of the multicolor LM‐LFIA. The immunoprobes were mixed and sprayed on the conjugate pad and the p72, E2 and GCIgY were sprayed as the T1‐line, T2‐line and C‐line, respectively, on the NC membrane. (C) Sample testing and result determination.

**Figure:**
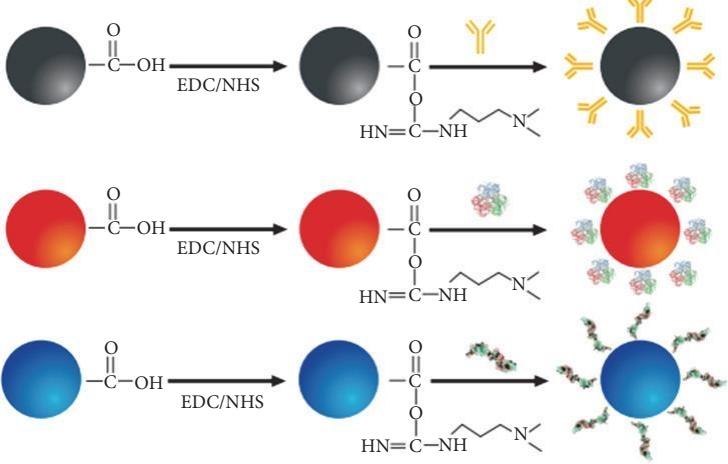
(A)

**Figure:**
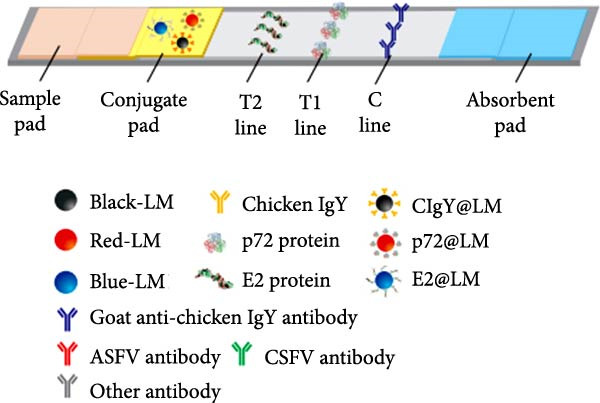
(B)

**Figure:**
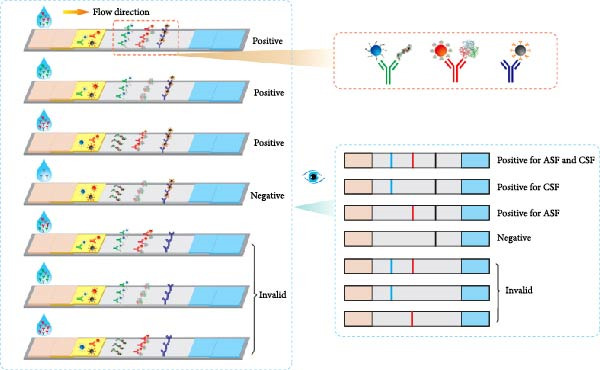
(C)

### 3.3. Characterization of the Immunoprobes

Dynamic light scattering (DLS) revealed a significant increase in hydrodynamic diameter upon protein conjugation. The average particle sizes increased from 297 ± 4.2 nm (RLM) to 505 ± 15.4 nm (p72@RLM) (Figure 3A), from 356 ± 8.1 nm (BLM) to 527 ± 9.5 nm (E2@BLM) (Figure 3B), and from 301 ± 6.9 nm (BkLM) to 542 ± 12.5 nm (CIgY@BkLM) (Figure 3C). Additionally, zeta potential measurements indicated significant changes in surface charge: shifting from −22.57 ± 2.94 mV (RLM) to −10.21 ± 0.39 mV (p72@RLM); −33.90 ± 6.19 mV (BLM) to −9.42 ± 0.89 mV (E2@BLM); and −28.57 ± 1.12 mV (BkLM) to −5.56 ± 0.66 mV (CIgY@BkLM) (Figure 3D). These findings confirmed the successful conjugation of proteins to LMs.

Figure 3Characterization of p72@RLM, E2@BLM and CIgY@BkLM. After conjugated with p72, E2 and CIgY, the properties such as particle size and surface charge of LM were examined by the dynamic light scattering. (A,B,C) Hydrodynamic size. (D) Surface zeta potential.

**Figure:**
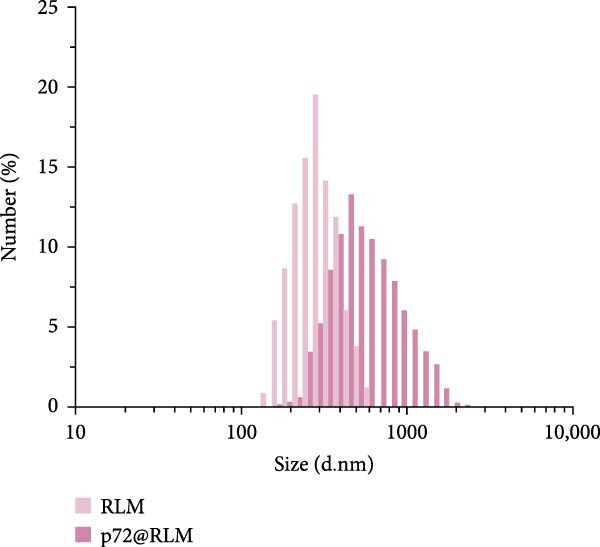
(A)

**Figure:**
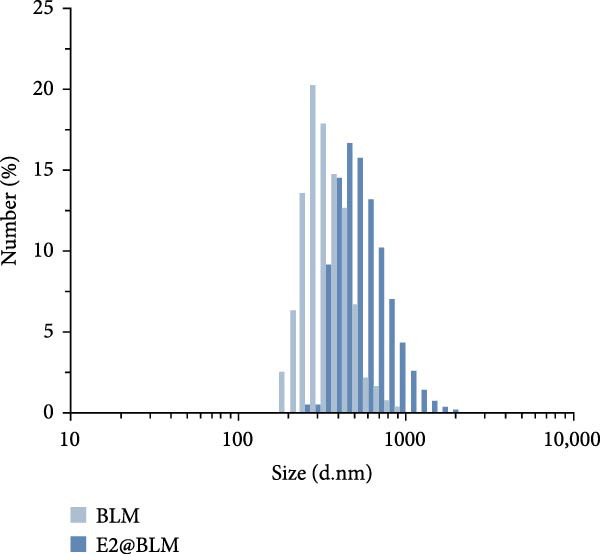
(B)

**Figure:**
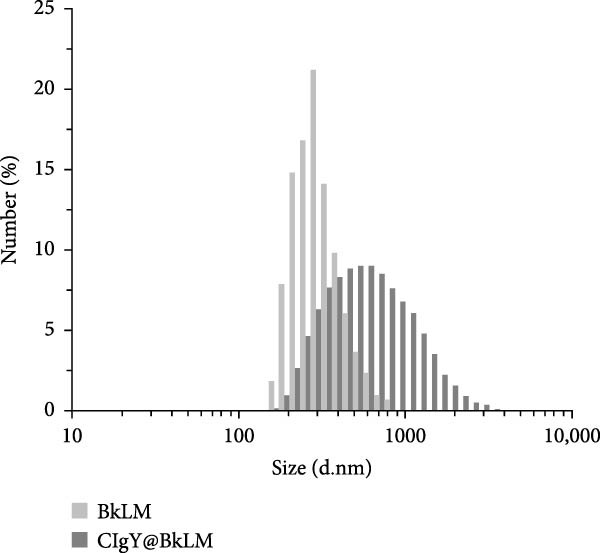
(C)

**Figure:**
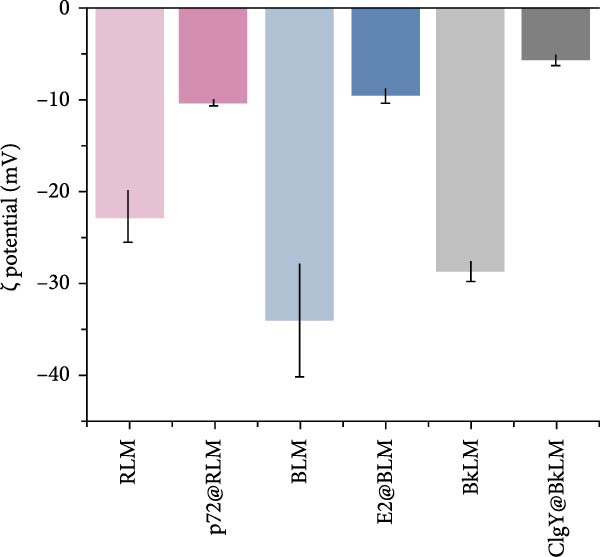
(D)

### 3.4. Optimization of Conjugation Parameters

To determine the optimal protein loading concentrations, LMs were conjugated with varying amounts (12.5–100 μg/mL) of p72, E2, and CIgY, respectively. Sedimentation and agglomeration were assessed at 5 min and 24 h postconjugation. For p72@RLM, a concentration of 50 μg/mL was identified as the optimal loading without significant aggregation (Figure 4A). Similarly, the optimal concentrations for E2 and CIgY were determined to be 50 and 62.5 μg/mL, respectively (Figure 4B,C).

Figure 4The optimal amount of protein conjugated with RLM, BLM, and BkLM. (A) RLMs were conjugated with different concentrations (12.5, 25, 37.5, 50, 62.5, 75, 87.5, and 100 μg/mL) of p72 at room temperature (RT). Sedimentation was observed after 5 min and 24 h of conjugation. The highest concentration that did not induce aggregation after 24 h was considered the optimal protein binding amount. (B,C), Similarly, BLMs and BkLMs were conjugated with different concentrations (12.5, 25, 37.5, 50, 62.5, 75, 87.5, and 100 μg/mL) of E2 and CIgY and then screened according to the above method.

**Figure:**
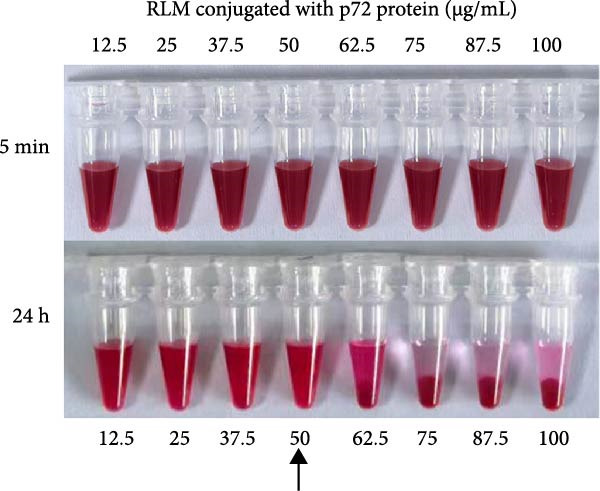
(A)

**Figure:**
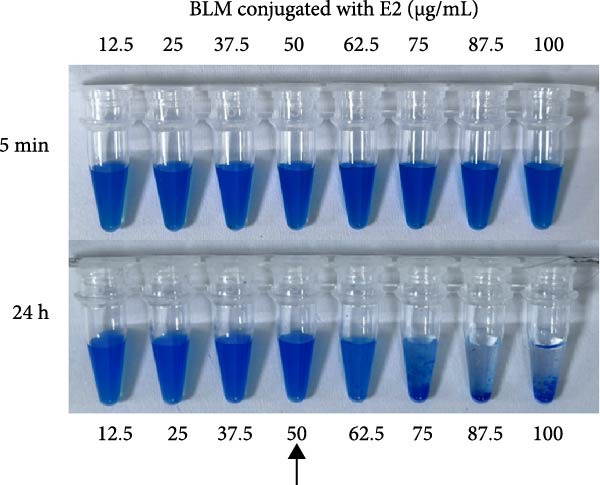
(B)

**Figure:**
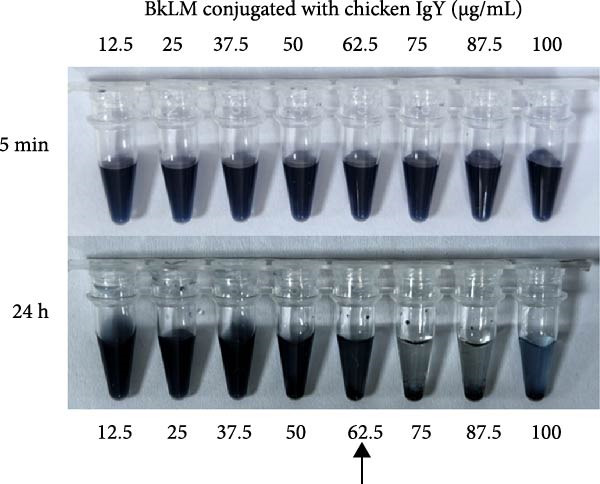
(C)

### 3.5. Performance Evaluation of the Multicolor LM‐LFIA

**Figure 6:**
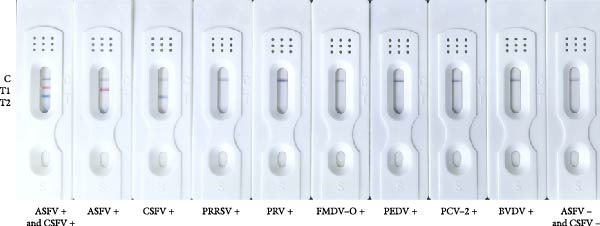
Specificity of the multicolor LM‐LFIA. ASFV‐positive and ‐negative serum and positive serum samples from common pathogens in swine such as CSFV, PRRSV, PRV, FMDV‐O, PEDV, PCV‐2, and BVDV were evaluated for specificity. No cross‐reactivity was noted, highlighting the strong specificity of this method. Three serum samples were tested for each pathogen, with each test repeated in triplicate.

Sensitivity: The sensitivity of the assay was evaluated using serially diluted standard positive sera (1:2 – 1:1024). The assay reliably detected ASFV and CSFV antibodies at a dilution of 1:256, with clearly visible T‐lines (Figure 5A). In comparison, after calculating the blocking rate according to their respective instructions, the commercial ELISA kits exhibited detection limits of 1:256 for ASFV and 1:64 for CSFV (Figure 5B), indicating that the multicolor LFIA is equally sensitive for ASFV and demonstrates a four fold improvement in sensitivity for CSFV. Specificity: no cross‐reactivity was observed when testing sera from pigs infected with unrelated pathogens, including PRRSV, PRV, FMDV‐O, PEDV, PCV‐2, and BVDV. Only ASFV‐ and CSFV‐positive sera yielded specific red or blue bands, respectively, demonstrating outstanding specificity (Figure 6). Repeatability: intrabatch and interbatch reproducibility were assessed using repeated tests on the same serum samples. All results were consistent across replicates and batches, with unambiguous identification of ASFV and CSFV antibody status based on T‐line color (Figure 7). These findings confirm the splendid reliability and batch‐to‐batch consistency of the assay.

Figure 5Sensitivity of the multicolor LM‐LFIA and commercial ELISA kits. The standard positive serum was serially diluted in PBS at a two‐fold ratio (from 1:2 to 1:1024), and each experiment was repeated in triplicate. (A) The sensitivity of LM‐LFIA was 1:256 based on the maximum dilution ratio of serum that generated visible T‐lines. (B) The same positive serum with each dilution was detected using commercial ELISA kits which are ASFV antibody detection and CSFV ELISA kits, the detection limits are 1:256 for ASFV and 1:64 for CSFV.

**Figure:**
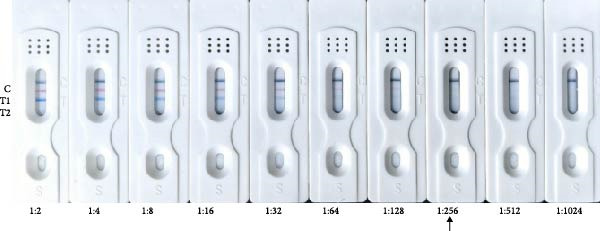
(A)

**Figure:**
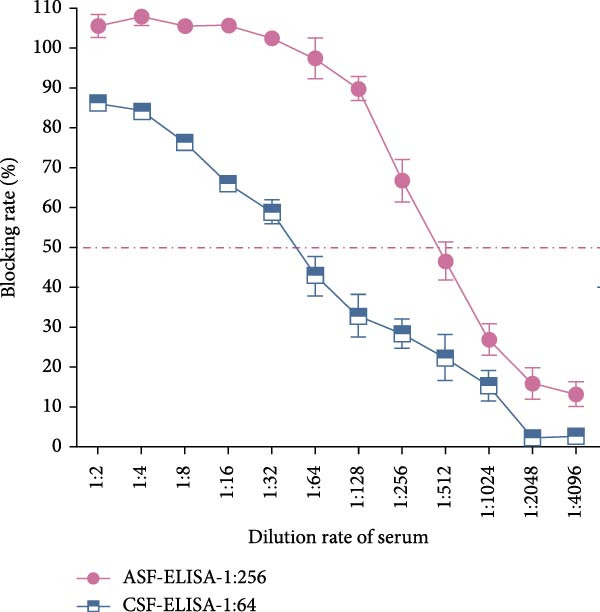
(B)

Figure 7Repeatability of the multicolor LM‐LFIA. (A) Intrabatch repeatability, the same sample was tested three times in parallel using LM‐LFIA from the same production batch. (B) Interbatch repeatability, to measure the same sample using test strips from three different batches, with each test repeated three times. A+, ASFV positive serum; A−, ASFV negative serum; C+, CSFV positive serum; C−, CSFV negative serum; C, control‐line; T, test‐line.

**Figure:**
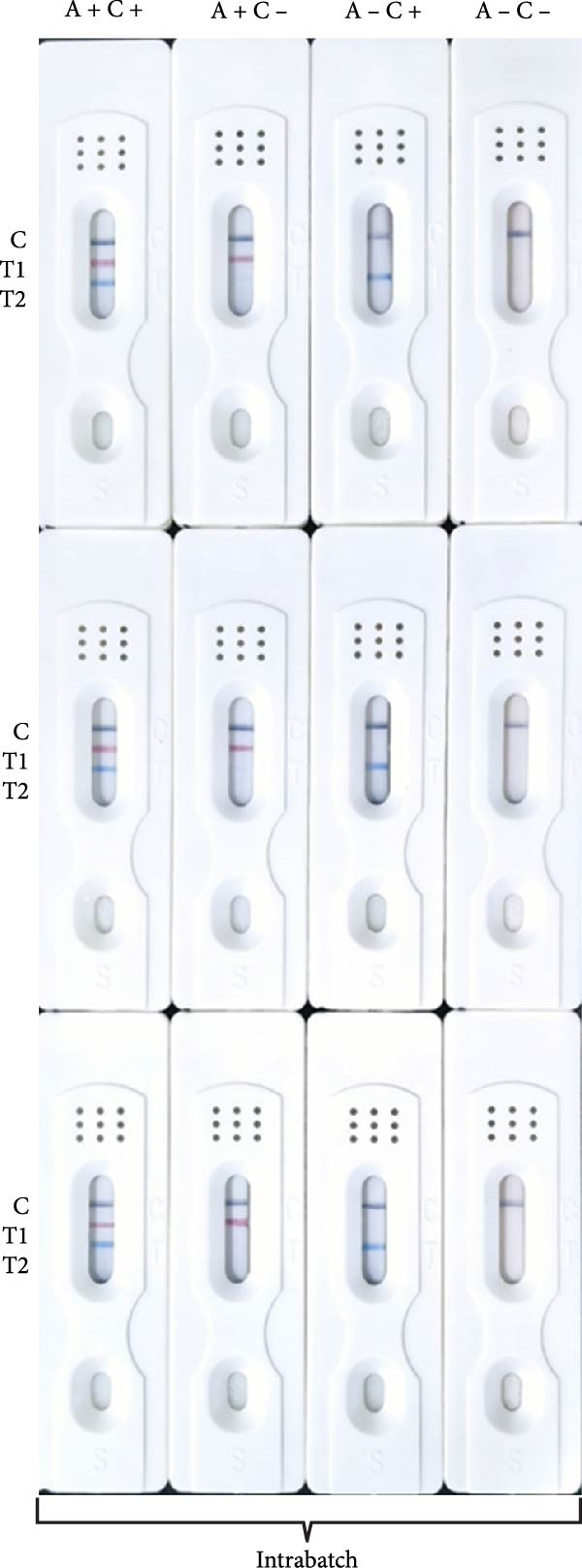
(A)

**Figure:**
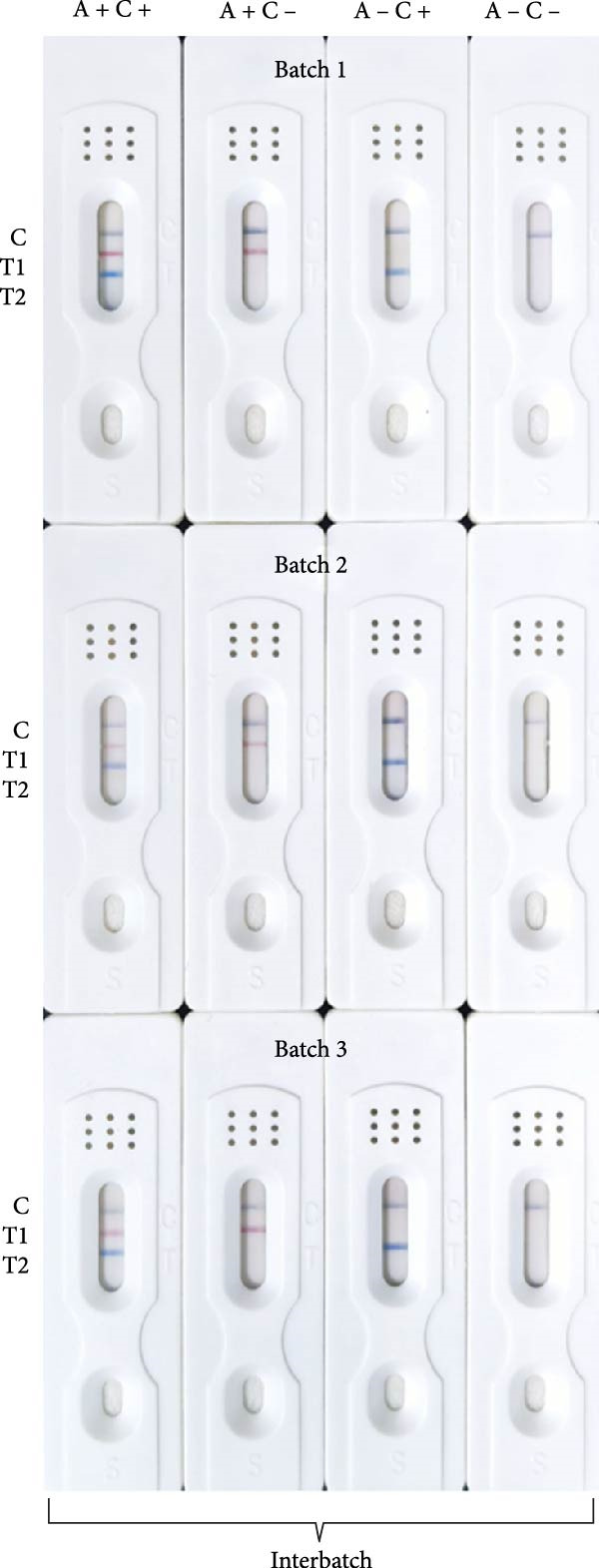
(B)

### 3.6. Clinical Application and Comparison With Commercial ELISA Kits

A total of 180 clinical serum samples were tested using the multicolor LM‐LFIA and benchmarked against commercial ELISA kits for ASFV and CSFV antibodies. The assay results showed high concordance with the ELISA kits, yielding Cohen’s kappa values of 0.986 for ASFV and 0.918 for CSFV (Table 1), indicating near‐perfect agreement and excellent diagnostic accuracy.

**Table 1:** Comparison of the multicolor LM‐LFIA and commercial ELISA kits for detecting ASF and CSF antibodies separately in clinical samples.

Multicolor LM‐LFIA	ELISA for ASF (Ingenasa)		Total	ELISA for CSF (IDEXX)		Total
	Negative	Positive		Negative	Positive	
Negative	131	0	131	65	2	67
Positive	1	48	49	5	108	113
Total	132	48	180	70	110	180
Kappa value		0.986			0.918	

## 4. Discussion

Both ASF and CSF are transboundary animal diseases of major economic and epidemiological concern and their clinical manifestations are often indistinguishable. Consequently, rapid identification and differentiation are essential for effective containment, especially in the absence of licensed vaccines for ASF [42]. In contrast, CSF can be controlled through vaccination, but outbreaks still occur due to vaccine failure, viral mutations, or immunosuppression from coinfections [43]. Hence, differential diagnosis remains a cornerstone of disease control strategies for both pathogens. Traditionally, diagnosis has relied on laboratory‐based assays, including virus isolation, qPCR, LAMP, and ELISA, all of which require specialized equipment, trained personnel, and central laboratory infrastructure. These limitations pose significant challenges in field conditions, particularly in resource‐limited or outbreak scenarios where timely decision‐making is critical [44]. The LFIA platform developed in this study directly addresses these limitations by enabling rapid, on‐site detection with minimal equipment and operational requirements [45].

The multicolor LM‐based LFIA introduced here represents a novel approach for the simultaneous detection and visual differentiation of antibodies against ASFV and CSFV. This platform integrates two key features‐multiplexing and visual discrimination‐into a single LFIA format. Red and blue LMs allow immediate identification of ASFV and CSFV antibody responses, respectively, while the black control line ensures assay validity. This color‐coded format [46] enables semi‐quantitative interpretation without the need for instrumentation, making it particularly suitable for pen‐side testing in field environments.

In terms of analytical performance, the assay demonstrated a sensitivity of 1:256 for both ASFV and CSFV antibodies, which is equivalent to or superior to that of leading commercial ELISA kits. Notably, the detection limit for CSFV antibodies was four fold higher than that of the commercial CSFV ELISA, highlighting the assay’s capability to detect low‐titer antibodies that might be missed by standard methods. Theoretically, high‐sensitivity antibody detection can help achieve early diagnosis. However, given the widespread vaccination of CSF vaccines, the diagnosis of CSF based on E2 antibodies is limited, which cannot achieve DIVA. This method is mainly used to evaluate the immune level and efficacy induced by vaccines, in this situation, positive results indicate protective antibody levels, while negative results indicate insufficient protection. In addition, the assay also exhibited excellent specificity, showing no cross‐reactivity with other common swine pathogens, including PRRSV, PRV, FMDV‐O, PEDV, PCV‐2, and BVDV, which belongs to the same genus as CSFV [47]. Repeatability testing confirmed consistent results across different batches, further supporting the assay’s robustness and manufacturing reliability. In clinical sample analysis, the multicolor LFIA showed strong agreement with commercial ELISA kits, with Cohen’s kappa values of 0.986 for ASFV and 0.918 for CSFV, indicating near‐perfect concordance. The slightly lower kappa for CSFV may reflect the higher sensitivity of the LFIA, allowing detection of low‐level antibodies undetected by ELISA.

Importantly, the use of multicolored LMs overcomes the conventional limitation of single‐analyte LFIAs. While earlier efforts in multiplex LFIAs often required spatially separated T lines or multiple devices [46], our approach achieves dual‐analyte detection in a compact, visually interpretable format. This design reduces sample volume, test time, as well as cost per sample. Specifically, the test requires only 10 μL of sample, delivers results within 15 min, and does not require separate tests or additional handling. Compared to standard ELISAs, which may take over 2 h and require skilled personnel, this LFIA provides a tenfold reduction in both detection time and sample volume. And, above all, the core application value of this multicolor LM‐LFIA lies in its ability to precisely implement the “teeth‐pulling” measure for ASFV when evaluating the immune effect of CSFV. Specifically, this multicolor LM‐LFIA is designed for the differential diagnosis between ASF and CSF, which means its practical application scenarios in various fields, including but not limited to ASF and CSF diagnosis in pig farms that have not been vaccinated with CSF vaccine, evaluation of immune efficacy of CSF vaccine in pig farms and differential diagnosis with ASF, as well as the on‐site testing during introduction and animal transportation in breeding farms, which can be conducted at any time without the need for any equipment. The success of this platform highlights the potential of LM‐based multiplex LFIAs in veterinary diagnostics. With appropriate modifications, this format could be extended to detect additional pathogens relevant to swine or even human health, such as PCV or *Mycoplasma hyopneumoniae* or COVID‐19. The scalability of LFIA manufacturing and the availability of diverse LM colors enable the development of high‐throughput, multitarget panels for broader surveillance applications.

In conclusion, the multicolor LM‐LFIA developed in this study offers a rapid, sensitive, specific, and user‐friendly tool for the differential diagnosis of ASF and CSF in field settings. It addresses key limitations of current methods and provides a practical solution for large‐scale serological surveillance. This work not only fills a critical diagnostic gap but also lays the foundation for future development of advanced multiplexed LFIAs for comprehensive veterinary and human disease monitoring. At present, this study can detect low‐level antibody conversion, but this result does not represent the relationship between antibody levels and immune protection rate, which may be the focus of our future research.

## Conflicts of Interest

The authors declare no conflicts of interest.

## Author Contributions

Jie Chen and Zhengwang Shi contributed equally to this study.

## Funding

This work was supported by National Key Research and Development Program of China (Grant 2021YFD1800100), The Open Competition Program of Top Ten Critical Priorities of Agricultural Science and Technology Innovation for the 14th Five‐Year Plan of Guangdong Province(Grants 2024KJ14,2023SDZG02), the STI 2030‐Major Projects (Grant 2023ZD0404301), Innovation Program of Chinese Academy of Agricultural Sciences, (CAAS‐CSLPDCP‐202302, CAAS‐ASTIP‐2024‐LVRI); the Science and Technology Major Project of Gansu Province(Grant 22ZD6NA012), China Agriculture Research System of Ministry of Finance and Ministry of Agriculture and Rural Affairs (CARS‐35), Science and Technology Plan Project of Gansu Province, (Grant 21IR7RA024), the Strategic Priority Research Program of Project of the National Center of Technology Innovation for Pigs,(Grant NCTIP‐XD/C03), and the Major Science and Technology Project of Gansu Province,(Grant 23ZDNA007).

## Data Availability

The data that support the findings of this study are available from the corresponding author upon reasonable request.
